# *Scoliosis*: a journal dedicated to multidisciplinary research on prevention, control, and treatment of scoliosis and other spinal deformities

**DOI:** 10.1186/1748-7161-1-1

**Published:** 2006-03-31

**Authors:** Hans-Rudolf Weiss

**Affiliations:** 1 Asklepios Katharina Schroth Spinal Deformities Rehabilitation Centre, Bad Sobernheim, Germany

*Scoliosis *is the brainchild of a dedicated group of researchers and clinicians. In September 2003 Dr. Manuel Rigo invited leading specialists on conservative treatment of scoliosis from all over the world to Barcelona, for the first International Conference on Conservative Management of Spinal Deformities. The meeting took place in January 2004 and has to be regarded as a great success. During this meeting the Study Group On Spinal Orthopaedic and Rehabilitation Treatment (SOSORT) was founded as a working group and it was decided to prepare a consensus meeting in Milan 2005. During this meeting, hosted by Dr. Stefano Negrini, a decision was made to establish the working group as a society with a formal website  and an official peer reviewed journal. This, in short, is the history of *Scoliosis*.

*Scoliosis *is dedicated to multidisciplinary research on prevention, control, and treatment of scoliosis and other spinal deformities. Scoliosis was described in the Hippocratic Collection (500 B.C.) and has clinical implications for a wide range of disciplines, including biomechanics, epidemiology, exercise physiology, physical therapy, orthopaedics, osteopathy, physiatry, psychology, and respiratory science. The following contributions are welcome: research, reviews, methodology articles, and case reports. The journal will also publish "technical notes" that focus on new technical developments in the field of physiotherapy, rehabilitation and orthotics.

*Scoliosis *is published by BioMed Central, an independent publisher committed to ensuring peer-reviewed biomedical research is open access. Articles will be freely and universally accessible online, and archived in several internationally recognized free access repositories, including PubMed Central, the US National Library of Medicine's full-text repository of life science literature, and repositories at the University of Potsdam in Germany, at INIST in France, and in e-Depot, the National Library of the Netherlands' digital archive of all electronic publications. Authors publishing in the journal retain copyright, allowing anyone to reproduce or disseminate articles, according to the BioMed Central copyright and license agreement.

*Scoliosis *will be a valuable resource in the field of conservative scoliosis therapy. The prevalence of mild to moderate scoliosis in adolescents is 3000–5000 per 100,000 population, and in adults as high as 12% [1,2]. Fortunately, only a small minority of cases (<1%) progress to a magnitude at which spinal fusion surgery is recommended [1,3]. Treatment indications for the remaining patients, as well as those individuals with severe scoliosis who decline surgery, have been a source of controversy [4,5]. Yet the lifetime disease burden for scoliosis patients has become increasingly clear [6-13]. Scoliosis is associated with increased pain in adults of all ages, compared with control populations [6,7]. Furthermore, children and adults with mild to moderate curvatures may have reduced vital capacity and exercise capacity [8-12], and young adults with moderate scoliosis exhibit measurable changes in cardiac function [13]. Recent surveys of the general population have revealed that deficits in respiratory function characteristic of scoliosis patients (<85% predicted for age, height, and gender) are strong predictors of cardiopulmonary disease and increased mortality [14-16]. Reduced respiratory function and increased pain may underlie the observation that scoliosis patients exhibit significantly impaired quality of life [17].

Treatment indications will be a primary focus for *Scoliosis*. In the last twenty years, with cooperation among the International Research Society for Spinal Deformities (IRSSD), increasing attention has been paid to scoliosis etiology and pathomechanism [18]. Careful examination for scoliosis, as well as screening policy established in some countries, has allowed early diagnosis of mild curvatures that can potentially be improved with nonoperative treatment. Early diagnosis has enabled physicians to propose physiotherapy and bracing in order to halt progression, and has resulted in a body of research consistent with the hypothesis that nonsurgical approaches can prevent progression and ameliorate signs and symptoms of spinal deformity [19,20]. Yet well-documented studies on conservative management of scoliosis are still needed in order to balance the therapeutic approach to this disease. As an example, among 2000 articles published on 'scoliosis' in the last 10 years (Medline, 1996–2005), more than 800 (40%) have focused on 'surgery' but only 20 (1%) on 'prevention and control.' *Scoliosis *will provide a forum to fill this gap, to facilitate international communication of professionals and, finally, to improve the care of patients living with scoliosis. Information gained through research on spinal deformities may also provide insight into pathological mechanisms underlying back pain which perennially afflicts a large proportion of the human population.

Each case of spinal deformity has its own 'natural history' with variable onset, progression, and symptoms [21], and informed patients need to be involved in the decision-making process regarding their own treatment. The open access policy of BioMed Central will foster access of patients, parents, pediatricians and others who need to be involved in the decision of when, whether, and how to treat, so that *Scoliosis *may develop as a forum for patients as well as professionals in the field of spinal deformities. I am very thankful to Stefan Busch and Tom Pollard from BioMed Central who have helped to get *Scoliosis *started, and to the members of the editorial board, who agreed to help me to keep *Scoliosis *on course for the future.

I am deeply indebted to the senior Board members of SOSORT, namely Theodoros B Grivas, MD; Tomasz Kotwicki, MD, Toru Maruyama, MD, Stefano Negrini, MD, Manuel Rigo, MD but also to Prof. Martha Hawes. Without their help it would have been impossible to establish *Scoliosis*.

I am honored by the task of acting as Editor-in-Chief of *Scoliosis*. It has been and will continue to be a great pleasure to work with all those professional and enthusiastic supporters whom I met on the way to the launch of our new journal.

Everyone involved wishes the journal a successful start. May we increase our knowledge, improve our skills and gain enthusiasm for the benefit of our patients.
